# GDP-Mannose 3,5-Epimerase: A View on Structure, Mechanism, and Industrial Potential

**DOI:** 10.3389/fmolb.2021.784142

**Published:** 2022-01-11

**Authors:** Koen Beerens, Ophelia Gevaert, Tom Desmet

**Affiliations:** Centre for Synthetic Biology (CSB)—Unit for Biocatalysis and Enzyme Engineering, Faculty of Bioscience Engineering, Ghent University, Gent, Belgium

**Keywords:** epimerase, GDP-mannose, NS-SDR, short-chain dehydrogenase/reductase (SDR), NDP-sugar active SDR (NS-SDR), L-sugar

## Abstract

GDP-mannose 3,5-epimerase (GM35E, GME) belongs to the short-chain dehydrogenase/reductase (SDR) protein superfamily and catalyses the conversion of GDP-d-mannose towards GDP-l-galactose. Although the overall reaction seems relatively simple (a double epimerization), the enzyme needs to orchestrate a complex set of chemical reactions, with no less than 6 catalysis steps (oxidation, 2x deprotonation, 2x protonation and reduction), to perform the double epimerization of GDP-mannose to GDP-l-galactose. The enzyme is involved in the biosynthesis of vitamin C in plants and lipopolysaccharide synthesis in bacteria. In this review, we provide a clear overview of these interesting epimerases, including the latest findings such as the recently characterized bacterial and thermostable GM35E representative and its mechanism revision but also focus on their industrial potential in rare sugar synthesis and glycorandomization.

## Introduction

Recently, l-sugars have attracted scientific and industrial attention due to their importance as key constituents of biologically relevant molecules (Xia et al., 2014; Hélaine et al., 2015) [e.g., in antibiotics (Yu et al., 2013), bioactive oligosaccharides (Mulloy and Forster, 2000)] and potential for the pharmaceutical industry as building blocks for antiviral and anticancer drugs (i.e., nucleosides analogues) (Mathé and Gosselin, 2006). An example of the latter is lamivudine (also known as 3TC), an l-nucleoside analogue that is used as antiretroviral medication to prevent and treat HIV/AIDS and treat chronic hepatitis B (Gumina et al., 2001). A main reason therefore lay in the altered properties of l-sugars (in comparison to their d-counterparts), such as advanced antiviral activity, ameliorated metabolic stability and/or favourable toxicological profiles (Ahmed, 2001; Beerens et al., 2012). Unfortunately, apart from some exceptions (i.e., l-arabinose, l-fucose and l-rhamnose), the majority of natural sugars occurs as the d-enantiomers, making it difficult to extract them from natural resources. Therefore, different (bio)chemical production processes have been evaluated aiming to increase l-sugar availability and allow their commercial exploitation (Ahmed, 2001; D’Alonzo et al., 2009; Beerens et al., 2012; Frihed et al., 2015). In recent years, researchers also tried to explore natural enzymes and (artificial) pathways to allow efficient production of l-sugars (Gevaert, 2020) *via* systems biocatalysis.

For this reason, GDP-mannose 3,5-epimerase (GM35E or GME, EC 5.1.3.18), which catalyses the reversible interconversion of GDP-d-mannose (GDP-Man **1**) towards GDP-l-galactose (GDP-l-Gal **2**), attracts special attention as it is one of the few enzymes that bridges between the abundant d-sugars and their rare l-counterparts and could hence be an important biocatalyst to contribute to the economic production of l-Gal and other l-sugars. Indeed, in addition to GM35E’s main product GDP-l-Gal, two other side products are also found in its reaction mixtures, namely GDP-l-gulose (GDP-l-Gul **3**) and GDP-d-altrose (GDP-d-Alt **4**) (Gevaert et al., 2020) (more info see below). Hence, the GM35E catalytic reaction paves the way for the synthesis of (nucleotide activated) l-Gal, l-Gul and d-Alt as well as derivatives and glycosides thereof. These include a diverse range of biologically relevant molecules such as antibiotics (Yu et al., 2013) and bioactive oligosaccharides (Mulloy and Forster, 2000). Examples thereof are bleomycin, an l-Gul containing glycopeptide antibiotic with antitumor properties produced by *Streptomyces verticillus* (Du et al., 2000; Yu et al., 2013) for which the sugar moiety has been shown to stimulates the uptake of this drug by cancer cells (Schroeder et al., 2014). In addition, both l-Gal and l-Gul show potential as building blocks of l-nucleoside-based antiviral and anticancer medications (Mathé and Gosselin, 2006; Woodyer et al., 2010). Furthermore, l-Gal is a constituent of some saponins (Gutiérrez et al., 2004) and other biopolymers (Delattre et al., 2011), which manifest various biological activities and consequently find applications in the food, agronomic, cosmetic, and pharmaceutical industry (Osbourn et al., 2011). On the other hand, d-Alt can be applied as a substrate in the synthesis of cyclic carbamates of derived glycosylamines (polymer chemistry) (Kovacs et al., 1995).

## History and Physiological Function

**FIGURE 1:**
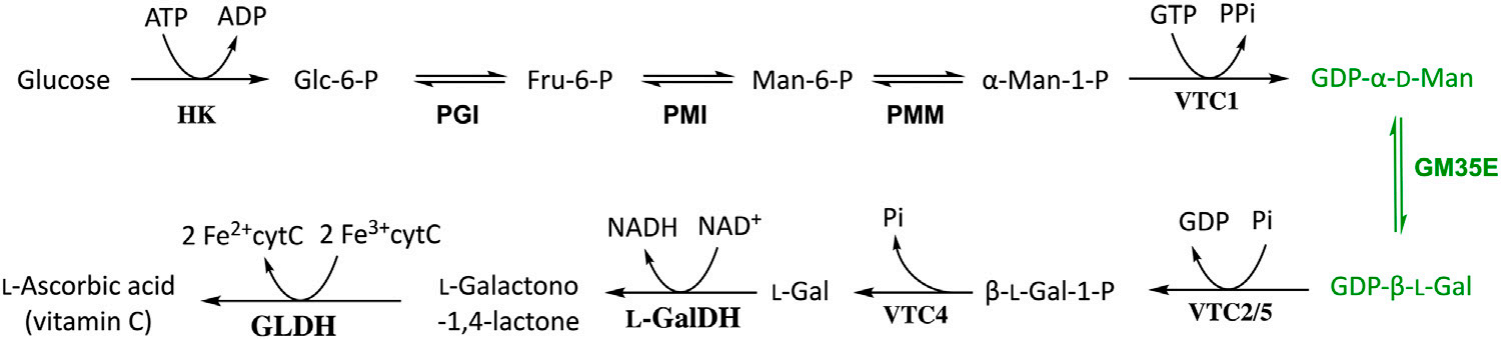
Schematic overview of l-ascorbate biosynthesis in photosynthetic organisms, known as the Smirnoff-Wheeler pathway, with the GM35E (GDP-Man 3,5-epimerase) highlighted in green. HK, hexokinase; PGI, phosphoglucoisomerase; PMI, phosphomannoisomerase; PMM, phosphomannomutase; VTC1, GDP-Man pyrophosphorylase; VTC2/5, GDP-l-Gal phosphorylase; VTC4, l-Gal-1-P phosphatase; l-GalDH, NAD^+^-dependent l-galactose dehydrogenase; GLDH, ferric cytochrome C-dependent l-galactono-1,4-lactone dehydrogenase.

The GM35E activity was first discovered over 50 years ago in the land snail *Helix pomatia* (Goudsmit and Neufeld, 1967), a decade later in freshwater green alga (*Auxeno*)*Chlorella pyrenoidosa* (Barber, 1979; Hebda et al., 1979; Barber and Hebda, 1982) and further research and understanding got a boost in the beginning of the 21^st^ century when GM35E homologues were discovered in plants (Wolucka et al., 2001; Wolucka and Van Montagu, 2003; Wolucka and Van Montagu, 2007; Major et al., 2005) and bacteria (Gevaert et al., 2019; Gevaert et al., 2020) (Table 1). The enzyme activity is linked to various functions in multiple organisms, such as agar and cell wall synthesis in algae (Barber, 1979; Hebda et al., 1979; Barber and Hebda, 1982) and production of polysaccharides and glycoconjugates in plants (Hebda et al., 1979; Watanabe et al., 2006; Delattre et al., 2011; Siow et al., 2013) as well as the synthesis of lipopolysaccharide (LPS) (Gevaert et al., 2019) and potentially even antibiotics (Du et al., 2000) in bacteria. *Arabidopsis thaliana* GM35E (*At*GM35E) is the most intensively studied GM35E (Wolucka et al., 2001; Wolucka and Van Montagu, 2003, 2007; Major et al., 2005), but also other homologues were evaluated for their role in the biosynthesis of vitamin C (l-ascorbic acid) in photosynthetic organisms (Wolucka et al., 2005; Watanabe et al., 2006; Gilbert et al., 2009; Voxeur et al., 2011; Yang et al., 2011; Zhang et al., 2011; Li et al., 2013; Chen et al., 2016) (Figure 1). Since vitamin C is an essential component to the human diet and it affects many crucial physiological processes (e.g., stress resistance and secondary metabolite biosynthesis) in plants, many studies focused on the elucidation of the vitamin C pathway and regulation (Wolucka and Van Montagu, 2003; Watanabe et al., 2006). These studies contributed to the understanding of GM35E’s physiological function in plants and algae, the characterization and investigation of plant-derived GM35Es (Wolucka et al., 2001; Wolucka and Van Montagu, 2003; Major et al., 2005; Wolucka et al., 2005; Watanabe et al., 2006; Wolucka and Van Montagu, 2007; Gilbert et al., 2009; Voxeur et al., 2011; Yang et al., 2011; Zhang et al., 2011; Li et al., 2013; Chen et al., 2016) and the first insights in its complex mechanism by crystal structure determination and mutagenesis (Major et al., 2005).

**TABLE 1:** Overview of biochemically characterized GM35E

Organism		Temp. opt. (range >50%)	pH opt. (Range)	K_M_ (µM)	V_max_ (µM min^−1^)	k_cat_ (s^−1^)	k_cat_/K_M_ (s^−1^ mM^−1^)	Structure^a^ (PDB)	Refs
(*Auxeno)Chlorella pyrenoidosa*	*Algae*	N.R.	8.1 (>80%: 7–9)	92 (GDP-Man) 97 (GDP-l-Gal)	2.8 (GDP-Man) 2.2 (GDP-l-Gal)	N.R.	N.R.	N.R.	Barber (1979), Hebda et al. (1979), Barber and Hebda (1982)
*Arabidopsis thaliana*	*Plantae*	N.R.	N.R.	4.5^a^ 18^b^ 31^c^ (GDP-Man)	1.76^a^ 0.31^b^ 0.43^c^ µmol h^−1^ mg^−1^	0.041^a^ 0.007^b^ 0.010^c^	9.1^a^ 0.4^b^ 0.3^c^	2C54, 2C59, 2C5A, 2C5E	Wolucka et al. (2001), Wolucka and Van Montagu (2003), Wolucka and Van Montagu (2007), Major et al. (2005)
*Oryza sativa*	*Plantae*	20–25°C	7.5–8.5	7.12 (GDP-Man)	N.R.	0.03	4.26	N.R.	Watanabe et al. (2006)
*Methylacidiphilum fumariolicum* strain SolV	Bacteria	60°C (45–70°C)	7.0–7.5 (>50%: 6.7–8.0)	98 (GDP-Man)	N.R.	0.2	2.04	N.D.	Gevaert et al. (2019), Gevaert et al. (2020)

a WT, or mutants in complex GDP-Man or GDP-l-Gal; N.R., not reported; N.D., not determined.

b Native enzyme purified from *A. thaliana*.

c Recombinant enzyme with N-terminal His-tag.

d Recombinant enzyme with N-terminal glutathione-S-transferase tag.

## Structure

Within the Carbohydrate Epimerase (CEP) classification (Van Overtveldt et al., 2015), GM35Es are clustered in the CEP1 family together with UDP-glucose 4-epimerases (GalE) (Beerens et al., 2013, 2015) and CDP-paratose/tyvelose 2-epimerase (CPa2E or TyvE) (Rapp et al., 2021). These CEP1 enzymes all belong to the large short-chain dehydrogenase/reductase (SDR) enzyme superfamily and more specifically the extended SDRs (Persson et al., 2009; Persson and Kallberg, 2013; Gräff et al., 2019). They share a common structural fold (i.e., extended Rossmann-fold with characteristic Glycine motif and catalytic triad ([ST]x_n_Yx_3_K) (Da Costa et al., 2021)) and mechanistic features (i.e., catalysis starts with an initial oxidation step, see below).

**FIGURE 2:**
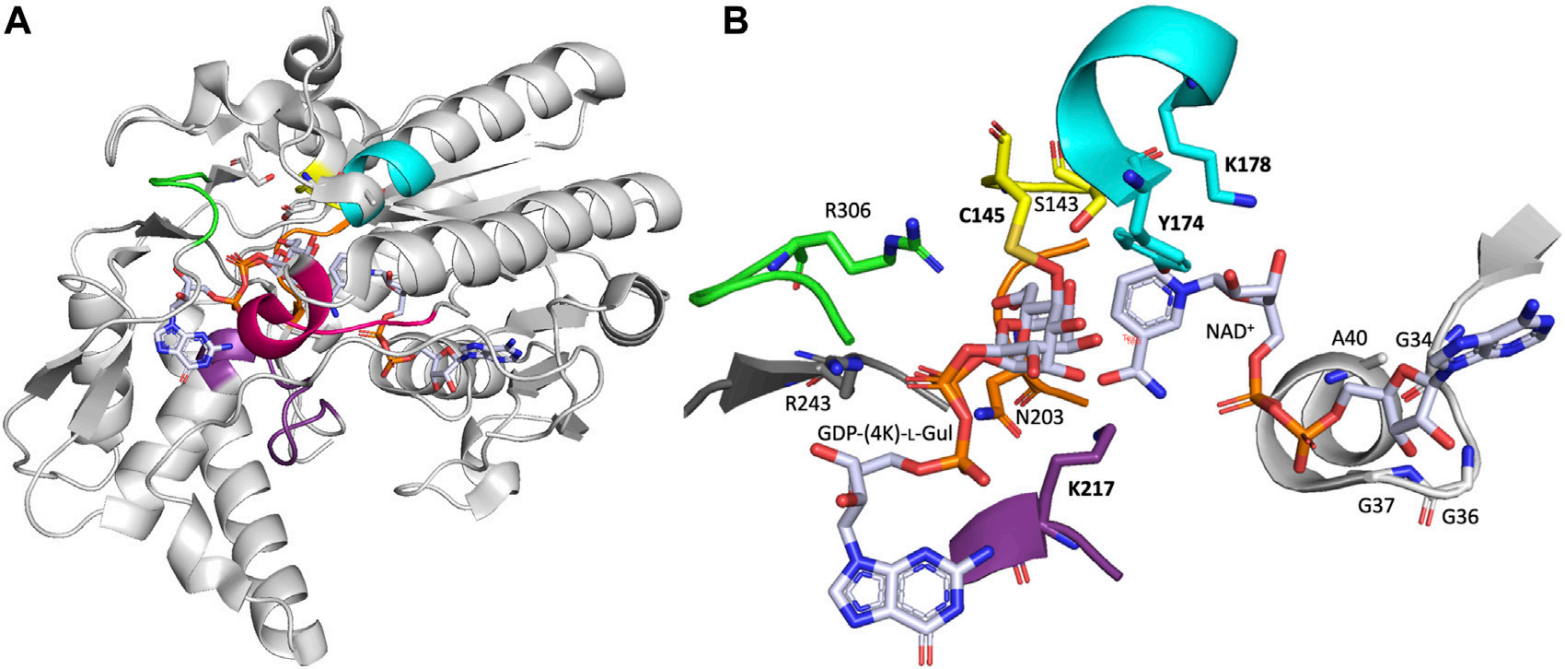
**(A)** One monomer of the crystal structure of *Arabidopsis thaliana* GM35E (*At*GM35E, PDB: 2C54) in complex with NAD^+^ and the reaction product GDP-l-Gul. **(B)** Zoom of the active site of *At*GM35E depicted by NAD^+^-binding Glycine-rich motif (light grey) and the walls of the heptagonal box model [in respective colors, for details see (Da Costa et al., 2021) and Figure 3]. The “Red wall” is not shown as it would block the view on the sugar and nicotinamide moiety of the NDP-sugar and NAD^+^, respectively. The most important residues are shown in sticks. These include the conserved Ser, Tyr and Lys from the catalytic triad ([ST]x_n_Yx_3_K) for C4-oxidation/reduction (S143, Y174 & K178), the Cys-Lys catalytic acid/base couple (C145 & K217), the C143-acidifying Arg (R306), the Arg that make an important interaction with the substrate’s di-phosphate backbone (R243) and the highly conserved Asn in the orange wall (N203).

Four crystal structure of native or inactivated variants of *Arabidopsis thaliana* GM35E (*At*GM35E) have been solved, which form a homodimeric quaternary structure and are complexed with substrate, products and/or reaction intermediate (Major et al., 2005). The monomeric structure of *At*GM35E in complex with GDP-l-Gul and GDP-4-keto-l-Gul (PDB code 2C54) will be discussed here in more detail (Figure 2). The crystal structure’s high resolution also allowed determination of the conformation of intermediates and thus helped to delineate the mechanistic possibilities of the epimerases (Major et al., 2005).

The *At*GM35E structure shows a Rossmann-fold domain, which binds one NAD^+^ cofactor per subunit, and a substrate binding domain to which the reaction product and intermediate are bound (Major et al., 2005). The organization of the secondary structure elements within the domains and the relationship between the domains is similar to that of other NS-SDR, like dTDP-glucose 4,6-dehydratase (RmlB) (Hegeman et al., 2001) and GalE (Beerens et al., 2015). Shortly summarized, GM35E binds NAD^+^ in a modified Rossmann-fold with seven parallel *β*-strands in its *β*-sheet flanked by three helices on each side (Figure 2). The Rossmann-fold has additional secondary structure elements that contribute to the substrate binding domain. The substrate binding domain is primarily helical with an antiparallel *β*-sheet and two short parallel *β*-sheets. Three loops from *At*GM35E’s C-terminus fold up against the Rossmann-fold. *At*GM35E forms a dimer with helices from one face of the Rossmann-fold forming the dimer interface. Superposition of the two monomers in the asymmetric unit shows that the N- and C-terminus as well as four loops differ by up to 2 Å for their C*α* positions. The substrate binding domain loops are also involved in crystal packing. Both N- and C-termini are flexible and when these regions were excluded a very good overlap was observed. The crystal structures of the variants and wild type, each containing different nucleotides sugars, are isomorphous. Structural analysis coupled to site-directed mutagenesis pinpointed C145 and K217 as the acid/base pair responsible for both epimerizations (for mechanism see below).

### Heptagonal Box to Correctly Identify and Annotate Novel GM35E

**FIGURE 3:**
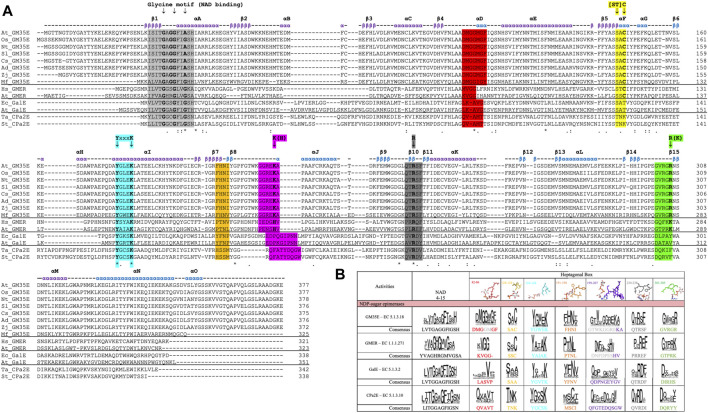
**(A)** Multiple Sequence Alignment (MSA) of several studied GM35E (At, *Arabidopsis thaliana*; Os, *Oryza sativa*; Nt, *Nicotiana tabacum*; Sl, *Solanum lycopersicum*; Cs, *Citrus sinensis*; Ad, *Actinidia deliciosa*; Zj, *Ziziphus jujuba*; Mf, *Methylacidiphilum fumariolicum* strain SolV) and 2 representatives of the related NS-SDR activities GMER, GalE and CPa2E (At, *Arabidopsis thaliana*; Hs, *Homo sapiens*; Ec, *Eschericia coli*; Ta, *Thermodesulfatator atlanticus*; St, *Salmonella typhi*), with highlighting of the Glycine motifs for NAD(P)^+^ binding (light grey), heptagonal box motifs (in corresponding colors) and important residues (arrows). The above given *α*-helices, *β*-strands and their numbering are from the *Arabidopsis thaliana* GM35E (*At*_GM35E). **(B)** Heptagonal box model of GM35E, GMER, GalE and CPa2E. The numbering of the motifs are derived from *Ta*CPa2E since this figure is adapted from Da Costa *et al.* (Da Costa et al., 2021).

Unfortunately, misannotations of GM35E as GalE or the related GDP-4-keto-6-deoxy-D-mannose-3,5-epimerase-4-reductase (GMER, also known as GDP-l-fucose synthase, GFS) (or vice versa) still occur for many automatic annotations of enzymes in databases. Our recent review and *in silico* analysis of NDP-sugar active SDR (NS-SDR) enzymes (Da Costa et al., 2021), also highlighted additional motifs and residues that can be applied to correctly identify and annotate novel GM35E. Inclusion of this heptagonal box model in the algorithms could improve the automated annotations. Indeed, GM35E and GMER clearly differ from GalE by their presence of an additional catalytic acid and base, which are needed for the double epimerization. The difference between GM35E and GMER is that they employ slightly different residues; the catalytic acid is in both cases a Cys (yellow wall), but the catalytic base is a Lys for the epimerase but a His for the epimerase-reductase (purple wall). In addition, also the residue in charge of acidifying the catalytic Cys is different, namely an Arg for GM35E and a Lys for GMER (green wall). They also show different Glycine motifs and different residues in between the catalytic Tyr and Lys (cyan wall). In addition, also the other three walls (red, purple and grey) of the heptagonal box model show to be slightly different for GM35E and GMER (Figure 3).

## Mechanism

**FIGURE 4:**
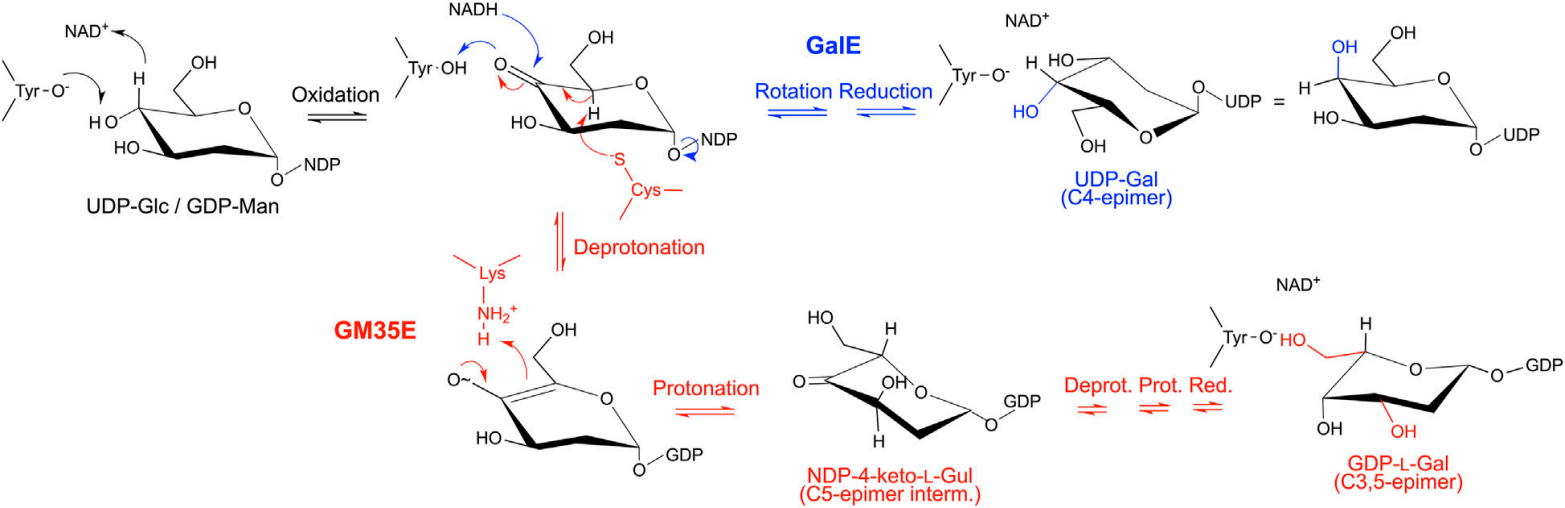
Mechanistic similarities demonstrate the evolutionary connection between GalE and GM35E. The mechanisms of both enzymes start with the formation of a keto-group at C4 (black), followed by a rotation and reduction of the keto-intermediate in GalE (blue), whereas an additional catalytic acid and base promote deprotonation/reprotonation at C5 and C3 in the GM35E (red) before final reduction of the keto-group at C4. The hydroxyl group at the C2 position is not shown for clarity reasons.

**FIGURE 5:**
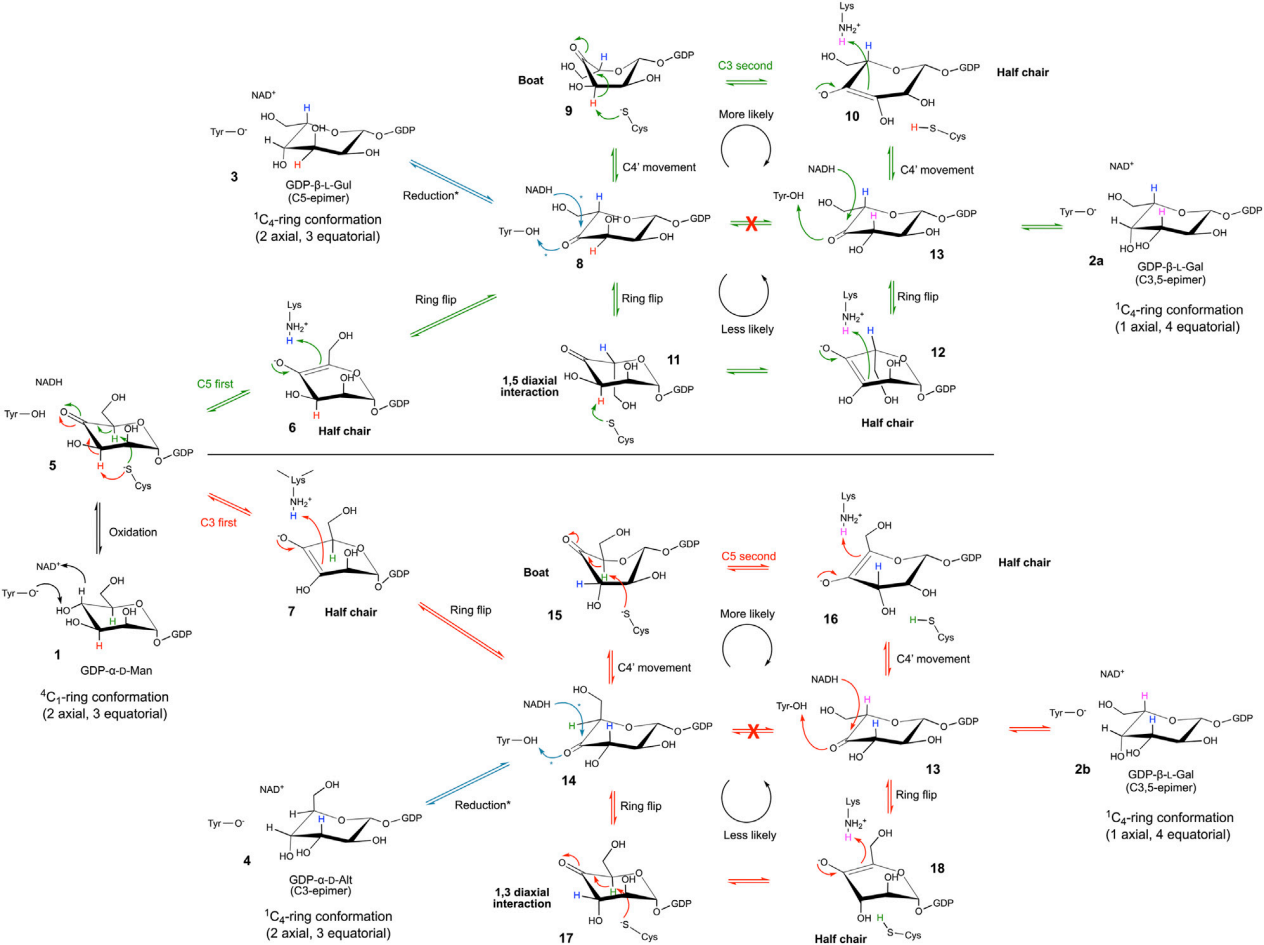
Mechanism of the GDP-mannose 3,5-epimerase. The epimerization reaction results in equilibrium between GDP-Man, GDP-l-Gal, GDP-l-Gul and GDP-d-Alt.

A key feature of the SDR superfamily, and thus also GM35E, is the transfer of a hydride between substrate and enzyme-bound NAD(P)^+^ cofactor. Indeed, it was found that the GM35E reaction starts with a C4-oxidation of GDP-Man **1** with the aid of the NAD^+^ cofactor, tightly bound to the enzyme, and the conserved Tyr (Yx_3_K) residue that acts as catalytic acid to assist the deprotonation, hereby highlighting its similarity to other CEP1 enzymes (Van Overtveldt et al., 2015). Instead of a simple rotation of the 4-ketopyranose intermediate and transfer of the hydride back to the C4 of the sugar, completing the 4-epimerization in GalE, the chemistry in GM35E is more complex (Figures 4, 5). Indeed, the initial oxidation results in a transient keto-intermediate at C4 (compound **5**), which merely functions to lower the pKa of the protons on the neighboring carbons (C3 and C5). Subsequently, an additional catalytic acid (Cys) and base (Lys) accomplish de- and reprotonation at both C5 and C3. The enolate that is created during this process is stabilized by hydrogen bonds with the catalytic Tyr, as well as with an additional Ser (S143) from the catalytic triad. Since initially only GDP-l-Gal **2** and GDP-l-Gul **3** (the C5-epimer of GDP-Man) were found in the reaction mixture, it was postulated that the enzyme performs the C5-epimerization prior to the C3-epimerization (Major et al., 2005) (Figure 4). However, GDP-d-Alt **4** was recently found as a reaction product, which means that both reaction routes can occur: C5-prior-to-C3 and C3-prior-to-C5 (Gevaert et al., 2020) (Figure 5). Eventually, the different keto-intermediate (GDP-4-keto-l-Gal **13**, GDP-4-keto-l-Gul **8** and GDP-4-keto-d-Alt **14**) can be reduced, resulting in its three reaction products, namely the main product GDP-l-Gal **2** (C3,5-epimer of GDP-Man) and two by-products: GDP-l-Gul **3** (C5-epimer) and GDP-d-Alt **4** (C3-epimer).

Initially, Major and coworkers took a closer look at the different reaction steps of the C5-prior-to-C3 route (Figure 5, green route) and predicted that a ring flip occurs during the first epimerization (C5) and that a boat intermediate is likely for the second epimerization (C3), suggesting a C4-movement prior to and after the second epimerization (Major et al., 2005). However, the observation of the GDP-d-Alt **4** as reaction product resulted in a detailed re-evaluation of these different reaction steps (Gevaert et al., 2020). Major and coworkers explained that for the C5-prior-to-C3 route, a sugar ring flip (compound **11**) would place the C3 proton in an axial position but would also place C6-O6 in an axial position, which was unlikely from earlier structural data. The ring-flipped conformation (compound **11**) would be significantly strained by a 1,5-diaxial clash between C6-O6 and O1. The second epimerization reaction would operate on a very high energy intermediate, and the transition state would have some 1,3,5-triaxial character, which is extremely unfavourable (compound **12**). Altogether this would present a formidable kinetic barrier to the second epimerization making this route much less likely, suggesting that the upper route with C4-movement (over a boat **9** and half chair **10** intermediate) is most plausible. A similar route is also most likely the route for C3-prior-to-C5-epimerisation. In this case, a ring flip would lead to disfavored 1,3-diaxial clash and 1,2,3-triaxial intermediate (compound **17** and **18**, resp.) again constraining the second epimerization by a kinetic barrier. Hence, also this route includes a C4-movement that is needed to position the substrate to allow the second proton abstraction at C5 (Figure 5, red route).

It is in fact not surprising that GM35E can utilize both routes to complete the overall double epimerization since it is proposed that the related GMER (full name: GDP-4-keto-6-deoxy-d-mannose-3,5-epimerase/4-reductase) catalyzes the C3,5-epimerization (of GDP-4-keto-6-deoxy-mannose) in a definite order, namely starting with C3-epimerization followed by the C5-epimerization before the reaction is completed by C4-ketone reduction, resulting in GDP-l-fucose (Rosano et al., 2000; Lau and Tanner, 2008). [This C3-prior-to-C5-epimerisation in GMER is based on the observation of the C3-intermediate in a crystal structure and the fact that the Cys109Ser mutant catalyzed a rapid wash-in of solvent derived deuterium into the C5 position of GDP-l-fucose in the presence of NADP^+^ (Rosano et al., 2000; Lau and Tanner, 2008)]. However, the confirmation that both routes to convert GDP-Man to GDP-l-Gal are possible for GM35E, combined with the structural and mechanistic similarity between GM35E and GMER, could also hint towards a dual epimerization route for GMER. Indeed, both enzymes employ very similar residues to achieve the double C3,5-double epimerization, namely Cys/Lys (Arg) and Cys/His (Lys). However, only detection of the GDP-4-keto-6-deoxy-l-gulose intermediate (C5-epimerization) or GDP-6-deoxy-l-gulose side product (C5-epimerization followed by reduction) in a GMER reaction could confirm this.

As mentioned by Major et al. GM35E are quite remarkable enzymes as they catalyze three distinct chemical reactions in one active site (i.e., oxidation, epimerization and reduction) and therefore a more correct name would be GDP-mannose 4-oxidase/3,5-epimerase/4-reductase, as it would better reflect the fact that they perform three distinct chemical catalytic reactions (Major et al., 2005).

## Industrial Potential of GM35E

### l-Galactose Production

**FIGURE 6:**
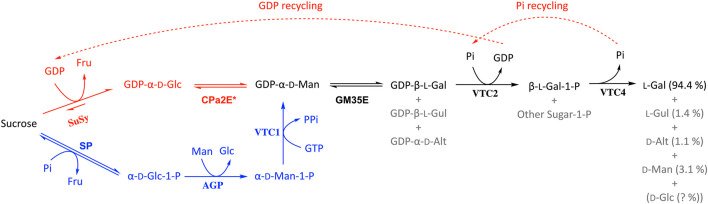
Biocatalytic pathway for the production of l-Gal starting from GDP-Man (as described in (Gevaert, 2020)) in which the GM35E plays a crucial role (black). The theoretical options to start l-Gal synthesis from sucrose are given in red and blue (explanation in main text). SuSy, sucrose synthase; CPa2E, CDP-paratose 2-epimerase; SP, sucrose phosphorylase; AGP, *α*-d-Glc 1-P phosphatase; VTC1, GDP-Man pyrophosphorylase; VTC2, GDP-l-Gal phosphorylase; VTC4, l-Gal-1-P phosphatase.

In terms of rare sugar production, epimerases play a key role as they can bridge between abundant d-sugars and their rare l-counterparts (Beerens et al., 2017) and this also holds true for the GM35E enzyme. Indeed, GM35Es (no matter their origin, microbial or plant) can theoretically be applied in a biocatalytic pathway to produce l-Gal, l-Gul and/or d-Alt. However, it has until now only been shown in practice for l-Gal starting from GDP-Man (Figure 6, in black) (Gevaert, 2020). After initial epimerization of GDP-Man to GDP-l-Gal (and side products GDP-l-Gul and GDP-d-Alt), the free l-Gal monosaccharide can be obtained by the addition of two other enzymes from the Smirnoff-Wheeler pathway. Starting from GDP-Man, Gevaert and co-workers achieved l-Gal with good yield (75%) and high purity (∼94%), due to the high selectivity of the additional enzyme(s) (Gevaert, 2020). Unfortunately, the GDP-Man used as substrate here remains a scarce and expensive substrate to produce l-Gal. Nonetheless, the Nidetzky group published an interesting pathway to produce GDP-Man from cheap sucrose and mannose in a kinase-independent one-pot multi-enzyme cascade (Pfeiffer et al., 2016), which could allow cheaper synthesis of GDP-Man (Figure 6, in blue). This setup for GDP-Man synthesis only requires one expensive NTP molecule (1 GTP), in comparison to the natural pathway from Glc or Man that requires 2 NTP molecules (1 ATP and 1 GTP) (Figure 1). Furthermore, another promising biocatalyst that could further improve GDP-Man (and thus l-Gal) synthesis is the recently discovered promiscuous CDP-paratose 2-epimerase (CPa2E), which is able to epimerise GDP-Glc to GDP-Man (Rapp et al., 2021). Hence, in combination with the sucrose synthase (SuSy) enzyme, this 2-epimerase could produce GDP-Man starting from sucrose and GDP (over GDP-Glc as pathway intermediate) (Figure 6, in red). Unfortunately, the CPa2E’s low activity currently remains a bottleneck. On the other hand, an additional benefit from this multi-enzyme pathway using SuSy and CPa2E is that the GDP released by the GDP-l-galactose phosphorylase can be recycled by the SuSy enzyme and thus requires only catalytic GDP quantities. In a similar manner the phosphate needed for the l-galactose 1-phopshate phosphatase would also be recycled (Figure 6, dashed arrows). However, implementation of the steps to produce l-Gal directly from sucrose remains to be investigated.

### Glycorandomization

**FIGURE 7:**
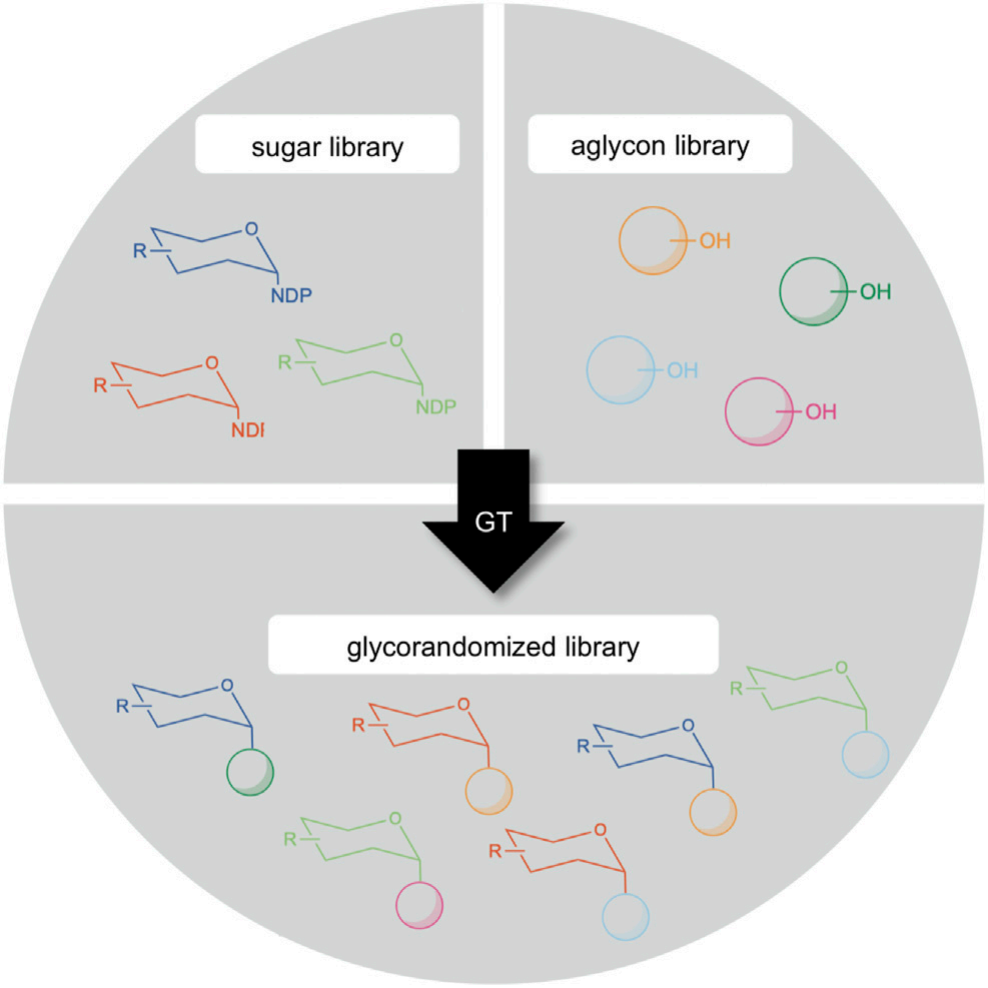
Creation of a glycorandomized library by glycosyltransferases (GT) through the diversification of sugar donor and/or acceptor (aglycon) molecule.

Another potential exploitation area of GM35E is situated in the technology called “glycorandomization” (sometimes also called “glycodiversification”, Figure 7). Since the majority of epimerases (such as GM35E) are active on NDP-sugars, their products can subsequently be valorized *via* for example glycosylation reactions. Glycosylation is the attachment of a carbohydrate (glycon) to another molecule (aglycon), yielding so-called glycosides. The aglycones can be very diverse and range from small molecules (e.g., vitamins, antibiotics, flavors and fragrances, etc.) to macromolecules (e.g., proteins, lipids and cell wall glycans) (De Bruyn et al., 2015; Desmet et al., 2012). Glycosides are widespread in nature and display a variety of interesting applications (Keegstra and Raikhel, 2001; Brandle and Telmer, 2007) and can show altered features compared than the free aglycon (e.g., increased water solubility or reduced chemical reactivity) but also based on the glycol attached (e.g., improved chemical stability or altered biological activity) (Jones and Vogt, 2001). Hence, glycosides can be applied in many industrial disciplines as for instance nutraceuticals, therapeutics or food additives (De Bruyn et al., 2015; Desmet et al., 2012). As pharmaceuticals, glycosides can both be used as an innovative approach for targeted drug delivery (Lepenies et al., 2010; Chen and Huang, 2019) or as an active moiety for therapeutic activity (Palcic, 2011). Furthermore, numerous antibiotics are glycosides and in many cases, the sugar entity is crucial to their activity and selectivity (Thorson et al., 2001; Kren and Rezanka, 2008; Song et al., 2013). For example, the antitumor antibiotic bleomycin contains a disaccharide moiety composed of l-Gul and 3-O-carbamoyl-d-Man, of which the l-Gul sugar unit possessed tumor cell targeting properties as it enables the uptake of the drug (Schroeder et al., 2014). Another example are macrolide antibiotics, such as erythromycin, which operate by binding bacterial ribosomes, thereby inhibiting protein synthesis. This binding is realized by their sugar moieties (Schroeder et al., 2014). Furthermore, also the l-Gal subunit of the nucleoside antibiotic A201A was found to be essential for the drug’s bioactivity (Zhu et al., 2017) and l-sugar containing analogs of the phytosteroid digitoxin showed an improved anti-cytomegalovirus activity (Cai et al., 2014). This effect of the carbohydrate moiety on therapeutics led to an increasing interest in unusual glycosides. On that account, glycorandomization has become a valuable tool for the expansion and optimization of various glycoside antibiotics. In this technology, the carbohydrate moiety of antibiotics is altered in order to tailor their pharmacological properties and/or biological activity (De Bruyn et al., 2015; Thibodeaux et al., 2007; Desmet et al., 2012). This approach facilitates drug screening and discovery and might be a powerful strategy in the battle against antibiotic resistance (Ventola, 2015; Mohs and Greig, 2017). Exotic NDP-sugar libraries can be created by a collection of epimerase specificities. These can be combined with aglycon libraries, consisting of both natural products and synthetic compounds, to generate novel glycorandomized libraries (Figure 7). The practice of the glycorandomization technology resulted in unnatural sugar analogues of vancomycin and the macrolide antibiotic YC-17 with improved antibacterial activity as compared to the natural product (Fu et al., 2005; Shinde et al., 2013). Moreover, erythromycin variants that regained activity against an erythromycin-resistant strain have been obtained (Zhang G. et al., 2015).

GM35E shows excellent capacity to make such NDP-sugar libraries as it would make a mixture of GDP-Man, GDP-l-Gal, GDP-l-Gul and GDP-d-Alt. Coupling to glycosyltransferases (GT, EC 2.4), which perform the majority of glycosylation reactions in nature (Desmet et al., 2012), is of course necessary since GTs catalyze the sugar transfer from a sugar donor to an acceptor molecule to generate glycosides with high efficiency and enantioselectivity (Lim, 2005). Within this large enzyme family, the Leloir glycosyltransferases use NDP-sugars as sugar donors (Lairson et al., 2008; Mestrom et al., 2019). GTs have been applied for the synthesis of natural glycosides and artificial derivatives (Luzhetskyy and Bechthold, 2008; Palcic, 2011), and have contributed to successful *in vivo* and *in vitro* glycodiversification projects (Blanchard and Thorson, 2006; Thibodeaux et al., 2008)*.* Despite most GT specificities discovered to date are highly specific towards both the donor and acceptor (Henrissat and Davies, 2000; Bowles et al., 2005), several promiscuous GTs have also been reported and could thus be used for glycodiversification. For example, SorF and OleD display flexible donor and acceptor specificities, respectively (Bolam et al., 2007; Kopp et al., 2007). In the case of OleD, its promiscuity is presumed to derive from its natural function as a defense mechanism against macrolide antibiotics. More interestingly, YjiC is a promiscuous GT with regard to both donor and acceptor molecules, spanning various chemical classes (Pandey et al., 2014). In addition, enzyme engineering already extensively contributed to the expansion of GT promiscuity (Williams et al., 2007; Jakeman, 2008; Williams et al., 2008; Chen, 2011; Gantt et al., 2011).

Similar as for several epimerases, the large-scale application of GTs is restricted by their need for NDP-sugars as substrates, which are expensive and hard to acquire in large quantities (Masada et al., 2007). Furthermore, it was suggested that NDP released during the transfer reaction can act as an inhibitor of GT activity, resulting in lower yields (Masada et al., 2007; Terasaka et al., 2012; Huang et al., 2016). Systems biocatalysis, the concept of creating cell-free “artificial metabolisms” for preparative multi-enzymatic synthesis (Fessner, 2015; Tessaro et al., 2015), might be applied to tackle these issues. Indeed, the use of regeneration systems allows the *in situ* regeneration of NDP-sugars, resulting in reduced costs and increased productivity (Ichikawa et al., 1994; Elleuche, 2015). Several of these systems including sucrose synthase (SuSy) have been developed to date, yielding for example glucosides of curcumin, resveratrol and quercetin (Masada et al., 2007; Terasaka et al., 2012; Lepak et al., 2015; Schmölzer et al., 2016). The NDP is recycled and the NDP-sugar regenerated by SuSy, yielding an overall reaction that would only require sucrose and catalytic amounts of NDP to proceed (Lim, 2005; Masada et al., 2007). Such cascade reaction could be expanded with epimerases (like GM35, but also CPa2E and/or GalE) to obtain various rare NDP-sugars as starting point for glycosylation, similar to the pathway shown in Figure 6, however, with promiscuous GTs at the end to achieve synthesize of a library of glycosides instead of releasing l-Gal. In a pathway, the equilibrium of the epimerization reactions is not an issue since the irreversible GT reaction pulls the overall cascade towards product formation.

### Importance and Application Potential of Plant GM35Es: Improved Stress Tolerance and Glycans Biosynthesis

Since l-ascorbic acid (also known as vitamin C) is an important plant antioxidant with important metabolic functions in both plants and animals (obtained *via* a plant-based diet), its plant biosynthesis has been studied intensively (Smirnoff and Wheeler, 2000) and plant engineering had already early on been suggested to achieve increased l-ascorbic acid production to increase both plant stress tolerance and nutritional value of humans and animals (Wheeler et al., 1998). Since both l-galactose (Wheeler et al., 1998) and l-gulose (Valpuesta and Botella, 2004) are important intermediates for l-ascorbic acid biosynthesis and both are derived from GDP-activated d-mannose, their synthesis is strongly dependent on the GDP-mannose-3,5-epimerase (and downstream enzymes). Hence, it is not surprising that GM35E and its pathway-connected enzymes have been studied in different plant species, including rice (Watanabe et al., 2006; Zhang G.-Y. et al., 2015), peach (Imai et al., 2009), cabbage (Ren et al., 2013), tomato (Mounet-Gilbert et al., 2016; Li et al., 2019), amongst others. Indeed, transgenic tomato plants over-expressing GM35E exhibited significantly increased l-ascorbic acid levels in leaves and red fruits compared with wild-type plants, which led to enhanced tolerance of different types of abiotic plant stress, incl. herbicide, oxidative stress, cold, and salt stress (Zhang et al., 2011). Similarly, transgenic GM35E overexpression in *Arabidopsis* also enhanced acid, drought and salt tolerance by increased l-ascorbate accumulation (Ma et al., 2014) and manipulation of the rice l-galactose pathway (incl. GM35E overexpression) also revealed enhanced salt stress tolerance (Zhang G.-Y. et al., 2015). However, this study by Zhang et al. (2015) suggest that another enzyme involved in the pathway, namely the GDP-l-galactose phosphorylase (GGP), may be a key rate-limiting step l-ascorbic acid biosynthesis, at least in rice. This, in fact, highlights that the entire l-ascorbic acid biosynthesis pathway is important and not only the GM35E. Indeed, studies with transgenic tobacco revealed that the equilibrium of the GM35E reaction is unfavorable to forward l-ascorbic acid biosynthesis, thus indicating a complex modulation (Imai et al., 2012). On the other hand, it has also been found that GM35E plays a key role beyond l-ascorbic acid, namely in non-cellulosic cell-wall biosynthesis. Indeed, GM35E silencing in tomato affected rhamnogalacturonan II (RG-II) structure (approx. 60% decrease in terminal l-Gal content in RG-II’s side chain A), hereby leading to a lower cross-linking capacity of the pectic polysaccharide RG-II and hindered normal plant growth and development (Voxeur et al., 2011). Similarly, earlier studies with transgenic tomato lines also showed growth defects affecting both cell division and cell expansion, as well as altered cell-wall monosaccharide content, especially mannose and l-galactose that is directly linked to GM35E activity, leading to plant fragility and loss of fruit firmness (Gilbert et al., 2009).

Considered all together, these findings highlight that GM35E activity is very important in plants, not only for l-ascorbic acid biosynthesis and the linked induced stress tolerance but also for its involvement in RG-II biosynthesis (Gilbert et al., 2009; Voxeur et al., 2011), needed for strong healthy plants and firm fruit. Hence, future applications of plant-derived GM35Es will most likely occur *via* plant engineering studies (transgenic overexpression) to enhance stress tolerance, obtain stronger plants and/or healthier and firmer fruit. Here, we recommend considering all enzymes involved in the l-ascorbic acid pathway instead of only the GM35E. Similarly, future studies focused on targeting RG-II biosynthesis should also focus beyond only GM35E and target the entire pathway.

### Production of l-galactosides: Interplay of GM35E and Promiscuous l-fucosyltransferases

In a similar fashion as described for the l-galactose production (as a monosaccharide) and glycans biosynthesis *in vivo* in plants, GM35E has also been applied for the synthesis of l-galactose-containing *N*-glycans *in vitro* (Ohashi et al., 2017). In this case, Ohashi et al. carried out a preparative scale GDP-l-Gal synthesis starting from GDP-Man by using recombinant *A. thaliana* GM35E followed by desalting, recycling high performance liquid chromatography and lyophilization steps. The produced and purified GDP-l-Gal was consecutively used as substrate for l-galactosylation, which was achieved using mouse *α*1,6-fucosyltransferase (*Mm*FUT8) or *A. thaliana α*1,3-fucosyltransferase (*At*FucTA) (Ohashi et al., 2017). Indeed, it had already been shown that multiple *α*-l-fucosyltransferases (FucT) were promiscuous towards GDP-l-Fuc analogs like GDP-l-Gal, leading to the formation of l-galactosylated Lewis structures (Stangier et al., 1997; Düffels et al., 2000). In these earlier cases, the GDP-l-Gal was synthesized either chemically or enzymatically using l-fucokinase/GDP-l-fucose pyrophosphorylase (FKP) rather than utilizing GM35E and starting from GDP-Man. The promiscuous nature of FucT has also been observed *in vivo* in the l-fucose deficient mur1 mutant of *A. thaliana* (Zablackis et al., 1996), in which l-galactose replaces the absent l-fucose as a required component of the biologically active cell wall xyloglucan-derived oligosaccharides. l-Galactosylation of these xyloglucan oligosaccharides by *At*FucT has recently also been demonstrated *in vitro* (Ohashi et al., 2019). *In vivo*, the GDP-l-Gal is most likely obtained *via de novo* synthesis over GDP-Man and thus utilizing the GM35E. Likewise, future production of other l-galactosides and l-galactose-containing glycans can utilize a combination of both GM35E and promiscuous l-fucosyltransferases. This setup is both achievable for biotransformations (*in vivo*) and biocatalytic production (*in vitro*). However, especially for the *in vitro* setup, it will be important to keep in mind the most efficient production route for GDP-l-Gal, either over GDP-Man using GM35E or methods starting from l-Gal directly.

Instead of using the GM35E’s major product (GDP-l-Gal), one could also aim at utilizing its minor side products (e.g., GDP-l-Gul). By using GTs that would (specifically) transfer l-gulose to acceptors, the same setup of GM35E coupled to GT could be used to produce l-gulosides. An example of a natural l-guloside is the main polar lipid of *Thermoplasma acidophilum*. Also here, the biosynthesis route for the activated l-gulose is expected to pass over an GM35E or homologous enzyme (Yamauchi and Nakayama, 2013). These natural sources of l-Gal and l-Gul could also be interesting targets to search for novel GM35E’s and/or GT enzymes that would be able to utilize GM35E’s products as substrate for glycoside production.

## Future Perspectives for GM35E (and Related Homologues/Enzymes)

To increase the portfolio of available GM35E biocatalysts that can be applied for industrial applications, an important research focus will be the characterization of novel GM35E homologues. This could be focused on bacteria and Archaea to obtain homologues with good recombinant overexpression and high (thermo) stability. Other important characteristics to look for are substrate promiscuity, for example variants that show activity on NDP-Glc, hence allowing synthesis of NDP-l-talose (C3,5), NDP-d-allose (C3) and NDP-l-idose (C5). Such enzymes would be interesting to both rare sugar and glycoside synthesis, and especially for glycorandomization studies. Pathway optimization will lead to increased yields and productivities. However, for the computational modelling of these pathways, it is important to perform in-depth characterizations (pH, temperature, selectivity/promiscuity, …) and detailed kinetic analysis of GM35E homologues (K_m_, k_cat_, inhibition, …) for the models to correctly predict and help to guide such pathway optimization studies.

**FIGURE 8:**
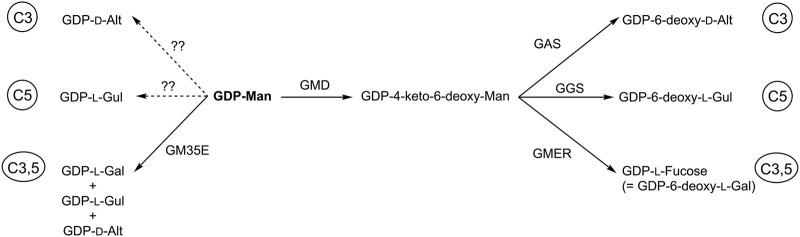
Conversion of GDP-mannose towards GDP-hexose epimers by GDP-mannose 3,5-epimerase (GM35E) or towards GDP-deoxy-hexoses by the consecutive action of GDP-mannose 4,6-dehydratase (GMD) and GDP-6-deoxy-d-altrose synthase (GAS), GDP-6-deoxy-l-gulose synthase (GGS) or GDP-4-keto-6-deoxy-l-mannose 3,5-epimerase-4-reductase (or GDP-l-fucose synthase, GMER).

GM35E belong to the CEP1 family, a group of epimerases displaying the same fold, similar mechanism and substrate, but are also part of the much bigger SDR superfamily. What can we learn from other CEP1 and/or SDR enzymes and what is applicable on GM35E? We recently reviewed and initiated *in silico* analysis of NS-SDR (short for NDP-Sugar active SDR enzymes (Da Costa et al., 2021)) in order to harvest information from the vast amount of NS-SDR and to guide engineering studies. An interesting observation is that where GM35E creates 3 products (GDP-l-Gal, GDP-l-Gul and GDP-d-Alt), the biosynthesis of the respective 6-deoxy-variants requires different specific bifunctional epimerase/reductase [after the initial GDP-mannose 4,6-dehydratase step needed for the deoxygenation), namely the GDP-4-keto-6-deoxy-d-mannose 3,5-epimerase/4-reductase (GMER also referred to as GDP-l-fucose synthase, GFS) (Rosano et al., 2000), a GDP-6-deoxy-l-gulose synthase (GGS, 5-epim.+4-red.)] (Galm et al., 2011) and a putative GDP-6-deoxy-d-altrose synthase (GAS, 3-epim.+4-red.) (Kudo et al., 2016) for GDP-l-Fucose (= GDP-6-deoxy-l-Gal), GDP-6-deoxy-l-Gul and GDP-6-deoxy-d-Alt, respectively. Like GM35E, these enzymes also belong the NS-SDR enzymes, once more highlighting the importance of NS-SDR for biosynthesis of special sugars but it also shows that similar enzymes perform either single (C3 or C5) or double (C3,5) epimerization reactions. Could this mean that also single NDP-sugar 3- and 5-epimerases exist in addition to the 3,5-epimerase? (Figure 8)? Such homologues are certainly worth screening for or could be targeted by enzyme engineering.

Shortly summarized, GDP-mannose 3,5-epimerase (GM35E) is a very special and interesting enzyme, initially mostly studied in plant research (where it remains highly relevant due to potential in improving stress tolerance for structural glycan synthesis), but has now found its way to applied biochemistry and systems biocatalysis: an interesting past and a challenging future ahead.
